# Genetic testing in cerebral palsy with clinical and neuroimaging variables

**DOI:** 10.1111/dmcn.16323

**Published:** 2025-04-05

**Authors:** Esther M. Tantsis, Shekeeb S. Mohammad, Simon P. Paget, Yisselle I. Virella‐Perez, Velda X. Han, Dianah Hadi, Chaya Goldman, Michelle A. Farrar, Michael Fahey, Russell C. Dale, the GENE‐CP study group, Kristine Alba‐Concepcion, David J Amor, Tajul Arifin Tajudin, Nadia Badawi, Elizabeth Barnes, Bruce Bennetts, Hilla Ben Pazi, Darius Ebrahimi‐Fakhari, Darcy Fehlings, Donna M Ferriero, Jennifer Friedman, Jozef Gecz, Gladys Ho, Sachin Gupta, Rod W Hunt, Kavitha Kothur, Michael Kruer, Manju A Kurian, Maria Kyriagis, Wang Tso Lee, Sarah McIntyre, Aurélie Méneret, Jonathan W Mink, Catherine Morgan, Angela Morrow, Nardo Nardocci, Emanuela Pagliano, Elizabeth E. Palmer, Toni S Pearson, Belén Pérez‐Dueñas, Emmanuel Roze, Michael Shevell, Anna te Velde, Mary‐Clare Waugh, Michèl A Willemsen, Yana A Wilson

**Affiliations:** ^1^ TJ Nelson Department of Neurology Children's Hospital Westmead Sydney NSW Australia; ^2^ Kids Neuroscience centre Sydney Children's Hospital Network Sydney Australia; ^3^ Department of Child and Adolescent Health, Faculty of Medicine and Health The University of Sydney Westmead NSW Australia; ^4^ Kids Rehab Children's Hospital Westmead Sydney NSW Australia; ^5^ Khoo Teck Puat–National University Children's Medical Institute National University Health System; Yong Loo Lin School of Medicine, National University Singapore Singapore; ^6^ Hospital Tunku Azizah Kuala Lumpur Kuala Lumpur Malaysia; ^7^ Paediatric Neurology Unit, Monash Children's Hospital, Department of Paediatrics Monash University Clayton VIC Australia; ^8^ Department of Neurology Sydney Children's Hospital Network Sydney NSW Australia; ^9^ Discipline of Paediatrics and Child Health, School of Clinical Medicine, UNSW Medicine and Health UNSW Sydney Sydney NSW Australia

## Abstract

**Aim:**

To optimize genetic testing in children with cerebral palsy (CP) by using clinical and magnetic resonance imaging (MRI) variables.

**Method:**

In this mixed methods study, we surveyed current approaches to genetic testing by Australian clinicians involved in the diagnosis of CP. Using an international expert panel we explored 78 variables, to determine which variables were thought to be supportive of monogenic CP. We tested the 78 variables in a retrospective cohort of 100 children with CP, of whom 21 had a genetic cause of CP.

**Results:**

Forty‐five clinicians replied to the survey of current practice, 91% agreed that genetic testing has a role in CP, although 47% thought that there was inadequate guidance on which patients to test. The expert panel reached 75% agreement for 30 out of 78 variables for genetic CP, and 14 out of 78 variables against a genetic cause of CP. Retrospective testing in 100 children with CP revealed dysmorphic features (odds ratio [OR] = 7.50; 95% confidence interval [CI] 1.88–29.85) and intellectual disability (OR = 4.86; 95% CI 1.29–18.30) were more common in those with genetic CP, and MRI being compatible with the clinical picture was the feature least common in genetic CP (OR = 0.14; 95% CI 0.05–0.41).

**Interpretation:**

Genetic testing has a role in determining CP aetiology; however, there is no consensus on who should be tested. We used mixed methodology and found that dysmorphic features, intellectual disability, and ‘MRI not compatible with the clinical picture’ are most supportive of a genetic cause of CP.

## Graphical abstract

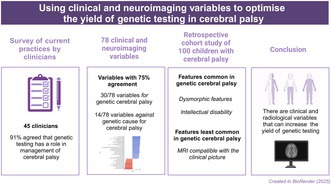

This original article is commented by Krägeloh‐Mann on pages 1376–1377 of this issue.

Plain language summary: https://onlinelibrary.wiley.com/doi/10.1111/dmcn.16334

**Abbreviation**
WES: whole‐exome sequencing

**What this paper adds**

- Genetic testing needs to be considered in the diagnostic work‐up of children with cerebral palsy.
- There are clinical and radiological variables that can improve the yield in genetic testing.
- These variables include dysmorphic features, intellectual disability, and ‘MRI not compatible with the clinical picture’.

Cerebral palsy (CP) is a lifelong disorder of movement and/or posture due to a non‐progressive aetiology. Monogenic causes contribute to aetiology, with a recently published meta‐analysis finding that 17.6% of children who had CP with no comorbidities (such as intellectual disability) had a genetic diagnosis, which increased to 37.8% in children with CP and intellectual disability.^1^ The benefits of genetic testing include potential for targeted therapies, better understanding and empowerment, connection to support groups, and reproductive choices.^2^ Historically selective CP cohorts such as those with normal imaging or no apparent cause (‘cryptogenic CP’^3^, ^4^, ^5^), have been shown to have a higher diagnostic yield on exome sequencing (42.1%; 95% confidence interval [CI] 36.0–48.2) compared with unselected groups (20.7%; 95% CI 12.3–30.5),^1^, ^4^, ^5^, ^6^, ^7^, ^8^, ^9^, ^10^, ^11^, ^12^, ^13^, ^14^ and 11.3% on a recent whole‐genome sequence analysis of 327 children.^15^

There is currently no consensus about which children with CP should be offered genetic testing, nor is there funding for testing in children based solely on their CP diagnosis in Australia and many other countries around the world.

An International Cerebral Palsy Genomics Consortium has been established with the goal of validating evidence‐based recommendations about genetic testing in individuals with CP, but a guideline is yet to be established.^16^, ^17^

One approach would be to offer all children with CP genetic testing,^18^ with a recent study showing that 8% of pathogenic/likely pathogenic findings in published genetic CP cohorts are actionable, and would prompt a change in clinical management related to the genetic aetiology.^19^ Another approach would be to establish selected parameters to refine testing and improve diagnostic yield,^20^ as has occurred in children with intellectual disability, where in Australia genetic testing is government funded for those with at least moderate intellectual disability. The diagnostic yield and relevance of testing must be assessed against the cost (time, psychological, and financial) in performing the testing itself. A recent economic evaluation has shown that the cost of a trio‐exome in Australia is USD3780.84 from sample collection to issuing a report, yet this cost does not factor in the time taken for a clinician and/or genetic counsellor to discuss results, generate clinic letters/support letters, or arrange counselling for other family members.^21^ Therefore, being selective of patients for testing remains a priority.

We performed a study to determine key aspects of genetic testing in CP. First, we surveyed clinicians to determine current practice for genetic testing in children with CP. Second, using an expert survey methodology we aimed to determine expert opinion on which clinical and investigation factors were predictive of a monogenic cause of CP. Third, we used the same clinical and investigation factors in a retrospective cohort of children with CP, comparing those having a confirmed genetic aetiology (pathogenic or likely pathogenic variant) with those considered to be ‘non‐genetic’ (either negative on testing, or not tested). The overarching aim of the project was to develop a clinical approach for improving genetic testing in children with CP.

## METHOD

### Clinician survey of current practice

We invited paediatricians (*n* = 50), neurologists (*n* = 22), rehabilitation specialists (*n* = 15), and neonatologists (*n* = 4) in the Sydney Children's Hospitals Network (Australia) with a minimum of 12 months' experience in the diagnosis and management of CP to participate in an online survey of current practice. A steering committee (ET, RCD, SM, MAF, MF) created a list of questions that were presented in an online format (Appendix S1). Clinicians were asked to vote on each statement of the questionnaire according to a 5‐point Likert scale (strongly agree/agree/neither agree nor disagree/disagree/strongly disagree) and provide open text comments as appropriate.

### Expert survey

We used an ‘expert panel’ approach to generate a list of factors that were proposed to predict a monogenic cause of CP. A steering committee (RCD, ET, SM, MAF, MF) generated a list of 78 clinical and radiological variables thought to be representative of the breadth of CP patient presentations (Table S1 and Appendix S2). The committee invited 29 international experts (‘the panel’) with representation from several continents and based on the individual: (1) being a specialist (usually paediatric neurologist, neuro‐geneticist, neonatologist, rehabilitation physician); (2) having a publication record in the field of CP including CP genomics; (3) being committed to completing the expert survey. There were 27 experts who responded in total: Australia (*n* = 10), North America (*n* = 7), Europe (*n* = 7), Asia (*n* = 2), and the Middle East (*n* = 1).

The panel members were asked to vote on each statement of the expert survey according to a 5‐point Likert scale (strongly agree/agree/neither agree nor disagree/disagree/strongly disagree) and provide open text comments as appropriate. All 78 variables were tested (Table S1). The survey responses were transformed into a ranked score system (+2 for strongly agree, +1 for agree, 0 for neutral, −1 for disagree, and − 2 for strongly disagree). Consensus was defined as an agreement by at least 75% of the participants (i.e. ≥75% agree/strongly agree or ≥75% disagree/strongly disagree).

### Retrospective review and testing of CP variables

Figure S1 shows the recruitment and breakdown of the retrospective cohort based on genetic testing. Following informed consent, the presence or absence of the clinical and radiological variables from the expert survey were analysed for all children from clinical notes, the original MRI, and supplemented by an interview with the family. All children were phenotyped using data elements as recommended by the International Cerebral Palsy Genomics Consortium Phenotype Working Group.^16^ As this was a retrospective review, clinical variables were determined as present or absent if they could be confirmed through assessments and clinical reports accessible in the medical record; for example, using the Bayley Scales of Infant and Toddler Development, Third and Fourth Editions for cognition. Imaging was initially analysed to determine patterns of injury using the variables from the expert survey (Table S1). One additional feature (not in the expert survey) was ‘MRI compatible with the clinical syndrome’. To rate this variable, the phenotyping and imaging data were reviewed, and a decision on whether the imaging was compatible with the clinical picture was scored dichotomously by the investigator (ET) blinded to genetic testing result. For the patient to have imaging compatible with the clinical presentation, the imaging was reviewed along with the clinical information (all data variables tested except genetic results). If the injury/damage/malformation could explain the impairment according to the clinical presentation, the imaging was scored as ‘compatible with the clinical syndrome’. Examples of MRI compatible and not compatible with the clinical syndrome are presented in Appendix S3. Genetic results were extracted directly from clinical reports.

### Statistics

A statistician (Elizabeth Barnes) was consulted before and during data analysis. Data were analysed using Microsoft Excel version 16.84 and SPSS version 28 (IBM Corp., Armonk, NY, USA). The mean scores of individual responses to the expert survey, along with the 95th CI, were visualized on a caterpillar plot, created with the ggplot2 package (version 3.4.4). For the retrospective cohort, features that were predictive of a genetic cause of CP were initially assessed in a univariable model using the risk option in SPSS. Risk was defined using odds ratio (OR) for genetic CP with each feature relative to those without the feature, with 95% CI and *p*‐value. All features predictive of genetic CP with a corrected *p*‐value of less than 0.05 on univariable analysis were then analysed using a multivariable model with binary logistic regression in SPSS. Initially, we identified and removed one variable that was highly correlated with another to reduce multicollinearity. The final model included 10 variables. To account for multiple comparisons, we applied the Benjamini–Hochberg correction using the STATS PADJUST extension. The model's goodness of fit was assessed using the Hosmer–Lemeshow test, and its discriminative ability was evaluated using the area under the receiver operating characteristic curve. The analyses were performed in two cohorts: in all children (*n* = 100) and in only those children for whom genetic testing was performed (*n* = 65).

### Ethics

Ethics approval was through the Sydney Children's Hospital Network. Approval numbers were 2021/ETH00595: Improving genetic testing in children with Cerebral Palsy (GENE‐CP) and 2022/ETH00462: Improving genetic testing in children with Cerebral Palsy (GENE‐CP) ‐ retrospective review and validation of score.

## RESULTS

### Australian clinician survey

**FIGURE 1:**
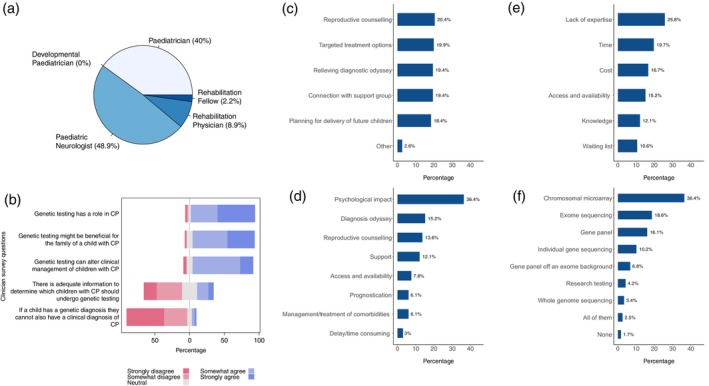
Clinician survey of current practice responses about the role of genetic testing in cerebral palsy (CP). In total, 45 clinicians responded to the survey of clinical current practice. (a) Breakdown of specialists who contributed to the survey with just under half of respondents being paediatric neurologists. (b) Most respondents agreed that genetic testing had a role in CP and that it would be beneficial to families. However, most felt that there was inadequate information to determine who should be tested. (c) The benefits of genetic testing according to our survey participants. (d) The perceived barriers to genetic testing as determined by the survey participants. (e) The perceived effect on families caused by a lack of access to genetic testing. (f) The types of genetic testing requested by our survey participants.

Figure 1 summarizes the main findings of clinicians' perspectives on genetic testing in CP. Forty‐five clinicians responded to the online survey (18 paediatricians, 22 neurologists, five rehabilitation physicians). Twenty‐five out of 45 (56%) of those surveyed had more than 10 years' experience. We collated the data by combining strongly agree with somewhat agree, and strongly disagree with somewhat disagree. Of responders, 48.9% were paediatric neurologists (Figure 1a). Forty‐one out of 45 (91%) responders agreed that genetic testing had a role in CP, and 39 out of 45 (87%) agreed that a genetic diagnosis did not negate a CP diagnosis. Twenty‐nine out of 45 (64%) clinicians agreed that there was benefit in genetic testing children with CP (Figure 1b); these benefits included reproductive counselling, connection with support groups, relieving the diagnostic odyssey, and targeted treatments (Figure 1c).

However, 24 out of 45 (53%) responders disagreed that there was adequate information to determine which children with CP should be genetically tested, and that the main barriers to genetic testing included lack of expertise, time, and cost (Figure 1b,e). The effect of a lack of access to genetic testing included psychological impact on families and perpetuation of the diagnostic odyssey (Figure 1d).

Thirty‐six out of 45 (80%) responders said that on average 15% of all children with CP undergo genetic testing under their care (range 5–25%). Of those surveyed, 15 (33%) thought genetic CP accounted for fewer than 20% of children with CP, and 22 out of 45 (49%) thought that genetic CP accounted for between 21% and 40% of children with CP.

Of the 45 respondents, 42 out of 45 used microarrays in their practice to investigate genetic causes of CP, 19 out of 45 used a gene panel, and 22 out of 45 used whole‐exome sequencing (WES) (Figure 1f). Access to genetic testing was reported to be difficult in 19 out of 45 (42%) of responders, whereas 14 out of 45 (31%) were equivocal, and a further 14 out of 45 (31%) thought access was easy. Thirty‐eight out of 45 (84%) responders agreed that genetic testing could result in a change of management (Figure 1b).

### Expert survey

**FIGURE 2:**
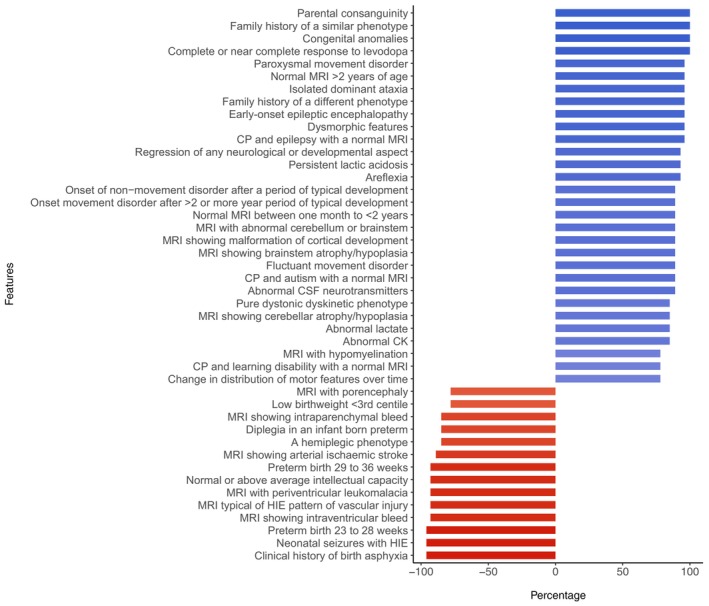
Expert survey factors likely to predict monogenic cause of cerebral palsy (CP) with greater than 75% agreement from the expert panel. There were 78 factors tested; 34 reached greater than 75% agreement. Factors in blue are thought to be more likely in those with genetic CP (*n* = 20), whereas factors in red are less likely in genetic CP (*n* = 14). Abbreviations: CSF, cerebrospinal fluid; HIE, hypoxic–ischaemic encephalopathy; MRI, magnetic resonance imaging.

Using our international expert panel, we examined which of the 78 clinical and radiological variables were most consistent or least consistent with genetic CP (according to expert opinion). In total, there was consensus with at least 75% agreement of the expert panel on 30 variables that were thought to be supportive of genetic CP, as shown in Figure 2 (blue). There were 14 variables with at least 75% agreement of the expert panel that were thought to be against a diagnosis of genetic CP shown in red in Figure 2.

### Examination of variables in a retrospective cohort of CP

Four hundred and twenty‐eight children with an established clinical diagnosis of CP were identified through current Sydney Children's Hospital Network Neurology and Rehabilitation Medicine clinics (a tertiary hospital for children). The diagnosis of CP was ascribed by general paediatricians, neurologists, or rehabilitation physicians, before recruitment. All families were given the study information and a follow‐up telephone call. Once we recruited our target of 100 children, recruitment ceased. The inclusion criteria for recruitment were a clinical diagnosis of CP (currently defined as a group of permanent, but not unchanging, disorders of movement and/or posture and of motor function, which are due to a non‐progressive interference, lesion, or abnormality of the developing/immature brain) regardless of risk factors or presumed aetiology (including genetic causes) and age less than 10 years (to ensure current standard of genetic testing). Exclusion criteria were age greater than 10 years and a progressive disorder not meeting criteria for CP. An MRI brain scan was performed in 95 of 100 children. Of the five children who did not have an MRI available for review, two had abnormal head ultrasound scans in the neonatal period, one patient declined imaging owing to the anaesthetic risk, one patient had an MRI brain overseas with only the report available (bilateral periventricular leukomalacia), and one patient was diagnosed with CP clinically and was awaiting MRI under general anaesthetic. In total, 65 children had genetic testing (microarray and/or WES). A microarray was performed in 60 children and a WES in 34 children. Twenty‐eight children had both a microarray and a WES performed. Five children had a WES but no microarray. No one had whole‐genome sequencing.

**FIGURE 3:**
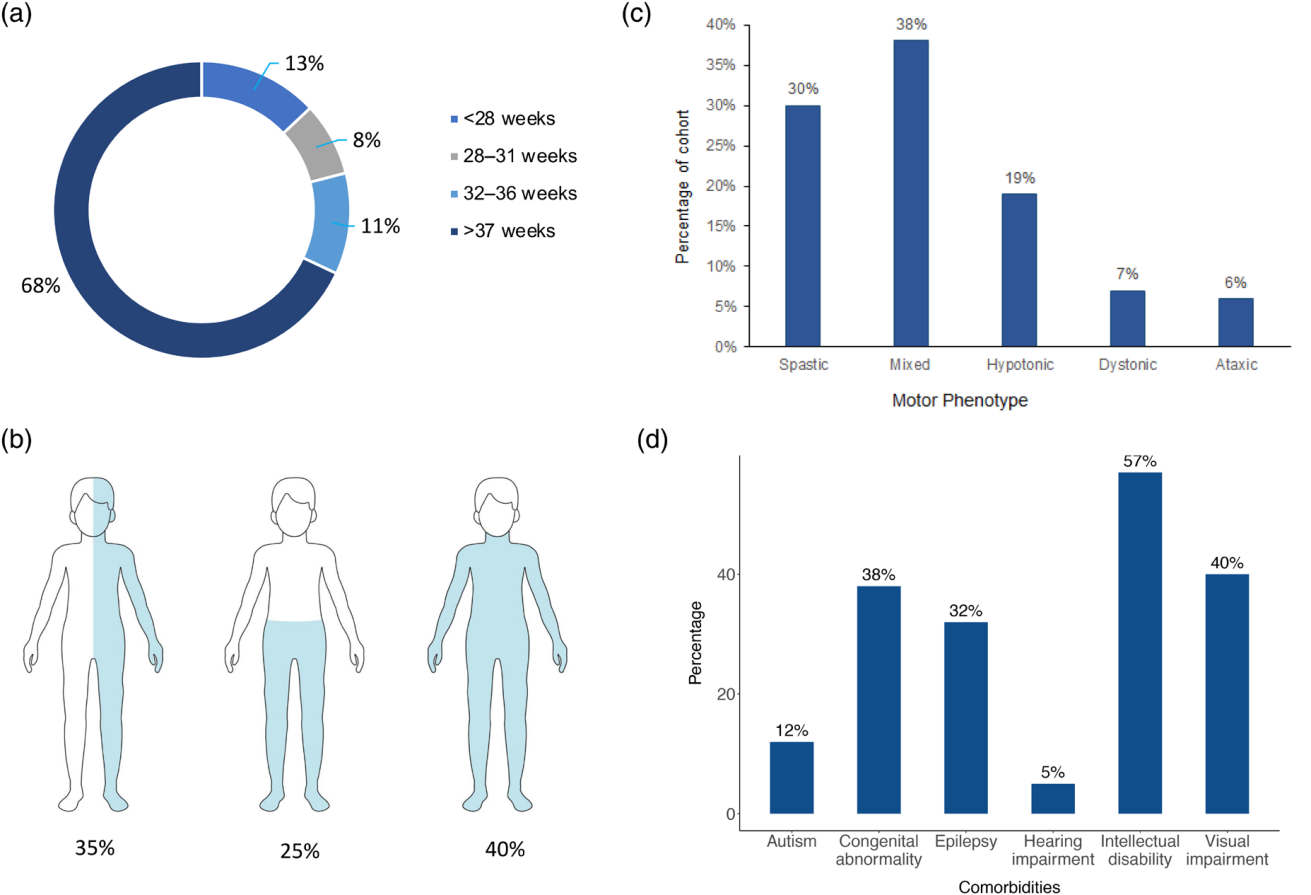
Demographics of retrospective cohort (*n* = 100). (a) Gestational age: 68% of the cohort were born at term. (b) Motor topography: 40% of our retrospective cohort had cerebral palsy with four‐limb involvement. (c) Motor phenotype: in our retrospective cohort, a mixture of dystonia and spasticity was most common and found in 38% of children. (d) Comorbidities: intellectual disability, visual impairment, congenital anomaly, and epilepsy were the most common findings.

Next, we took the same 78 variables used in the expert panel and tested them in a cohort of 100 sequential children with CP (Table S1 and Figure S2). Of the 100 recruited children, 64 were males and 68% of the children were born after 37 weeks' gestation (full term) (Figure 3a). The median age of the cohort was 6 years (range 6 months–10 years). The motor topography, phenotype, and comorbidities of the cohort are presented in Figure 3b–d.

A pathogenic or likely pathogenic variant was present in 7 out of 60 children tested by microarray and an additional seven children had a variant of unknown significance. A pathogenic or likely pathogenic variant was present in 14 out of 34 children tested by WES and a further six had a variant of unknown significance. In total, 21 children had a variant by either microarray or WES and were therefore designated as having genetic CP versus 79 with non‐genetic CP (negative or variant of unknown significance genetic testing, or no genetic testing).

Table S2 summarizes the gene findings of children who had pathogenic or likely pathogenic mutations (*n* = 21) on testing.

In total, 18 out of 95 (19%) children had a normal MRI brain scan: a normal MRI was present in 9 out of 21 (43%) with genetic CP versus 9 out of 78 (12%) with non‐genetic CP. All the children with non‐genetic CP and a normal MRI brain scan (*n* = 9) had genetic testing, including a microarray (*n* = 9) and an exome (*n* = 4).

All 78 clinical and radiological factors, including those with 75% agreement in the expert survey, were assessed in the retrospective cohort; the factors that demonstrated an odds ratio with 95% CI below or above 1 on univariable analysis are outlined in Table 1. None of the factors retained significance after multivariable analysis. The factors were assessed in the whole cohort (Table 1) and then just in the children who had genetic testing as part of their clinical work‐up (Table 2). The most significant factor supportive of a genetic cause of CP was dysmorphic features in the total cohort (Table 1), and the genetically tested only cohort (Table 2). The factors that were most against a genetic cause of CP were MRI compatible with the clinical picture (total cohort), and sequential MRI showing atrophy (genetic testing only cohort). ‘MRI compatible with clinical picture’ was significantly against a diagnosis of CP in the total cohort as well as in the genetically tested only cohort (Tables 1 and 2).

**TABLE 1:** Retrospective cohort: clinical and radiological factors predictive of genetic CP on univariable analysis in the whole cohort (21 with a genetic cause vs 79 without or no gene testing), presented as odds ratio and confidence intervals.

	Genetic CP, *n* (%)	Non‐genetic CP, *n* (%)	OR; 95% CI	*p*	Adjusted *p* ^a^
**Positive significant factors**					
Dysmorphic features	6 out of 21 (29)	4 out of 79 (5)	7.50; 1.88–29.85	0.004	0.02
Intellectual disability	15 out of 18 (83)	35 out of 69 (51)	4.86; 1.29–18.30	0.020	0.04
Small head circumference (<3rd centile)	9 out of 21 (43)	11 out of 77 (14)	4.50; 1.54–13.18	0.006	0.02
MRI showing malformation of cortical development	6 out of 21 (29)	7 out of 74 (9)	3.83; 1.12–13.04	0.032	0.04
Regression (loss of skills) of any neurological/developmental aspect	10 out of 21 (48)	16 out of 79 (20)	3.58; 1.29–9.90	0.014	0.04
Hypotonia/floppy	15 out of 21 (71)	34 out of 79 (43)	3.31; 1.16–9.42	0.025	0.03
Worsening of symptoms with fasting/illness	13 out of 21 (62)	29 out of 79 (37)	2.80; 1.04–7.56	0.042	0.04
**Negative significant factors**					
A hemiplegic phenotype	3 out of 21 (14)	32 out of 79 (41)	0.24; 0.07–0.90	0.034	0.04
Normal or above average intellectual capacity	3 out of 18 (17)	33 out of 69 (48)	0.22; 0.06–0.82	0.024	0.04
Preterm 23–36 weeks	1 out of 21 (5)	30 out of 79 (38)	0.17; 0.04–0.79	0.024	0.04
The MRI is compatible with the clinical picture	8 out of 21 (38)	60 out of 74 (81)	0.14; 0.05–0.41	<0.001	0.003

*Note*: Factors are presented in descending order of odds ratio.

Abbreviations: CI, confidence interval; CP, cerebral palsy; OR, odds ratio.

^a^ The *p*‐values were adjusted for multiple comparisons using the Benjamini–Hochberg correction.

**TABLE 2:** Retrospective cohort: clinical and radiological factors predictive of genetic CP on univariable analysis in those patients who had genetic testing (*n* = 65).

	Genetic CP	Non‐genetic CP	OR; 95% CI	*p*	Adjusted *p* ^a^
**Positive significant factors**					
Dysmorphic features	6 out of 21 (29)	3 out of 44 (7)	−5.47; 1.21–24.67	0.027	0.189^b^
Intellectual disability	15 out of 18 (83)	19 out of 37 (51)	−4.74; 1.17–19.16	0.029	0.05
Regression (loss of skills) of any neurological/ developmental aspect	10 out of 21 (48)	9 out of 44 (20)	−3.54; 1.15–10.91	0.028	0.1^b^
Small head circumference (<3rd centile)	9 out of 21 (43)	8 out of 44 (18)	−3.38; 1.06–10.71	0.039	0.04
**Negative significant factors**					
The MRI is compatible with the clinical picture	8 out of 21 (38)	29 out of 43 (67)	−0.30; 0.10–0.88	0.029	0.07^b^
Normal or above average intellectual capacity	3 out of 18 (17)	18 out of 37 (49)	−0.21; 0.05–0.85	0.029	0.04
Sequential MRI showing atrophy of any brain region	2 out of 7 (29)	11 out of 14 (79)	−0.11; 0.01–0.87	0.037	0.04

Abbreviations: CI, confidence interval; CP, cerebral palsy; OR, odds ratio.

^a^ The *p*‐values were adjusted for multiple comparisons using the Benjamini–Hochberg correction.

^b^ These factors were retained after adjusting the *p*‐values as the odd ratios were still indicative of a positive or negative predictive trend for genetic CP with 95% CI either >1 or <1.

## DISCUSSION

According to recent studies, genetic CP accounts for between 11.3%^15^ and 34.8% of children with CP.^1^ Most current practice guidelines suggest neuroimaging be performed early and genetic testing be reserved for those with a progressive course, normal neuroimaging, dysmorphic features, and/or consanguinity^15^, ^22^, ^23^ but this is probably missing a significant number of children with monogenic CP which may have implications for the child, family, and clinicians (therapeutic pathways).^20^, ^24^ Certain genetic findings may inform precision therapies such as l‐serine or memantine in *N*‐methyl‐d‐aspartate (NMDA) receptor variants (*GRIN1*, *GRIN2A*, etc.), or the choice of repurposed medications for symptomatic benefit such as 4‐aminopyridine or acetazolamide in *CACNA1A*, caffeine in *ADCY5*, Janus kinase (JAK) inhibitors in interferonopathies, and deep‐brain stimulation for dyskinetic crises in certain genetic disorders such as *GNAO1*, *UBA5*, *KMT2B*, and others.

From our survey of current practice, it is evident that most clinicians who diagnose and manage CP think genetic testing has a role in the work‐up of CP, but that adequate guidelines on who should be tested have not been established.

There were 44 factors that CP experts agreed to be supportive (30 factors) or against (14 factors) genetic CP (using 75% consensus) from a total of 78 clinical and radiological factors proposed. Fifteen of these 44 factors related to neuroimaging findings which supports the importance of MRI in the CP diagnostic work‐up.

When we tested all 78 clinical and radiological factors in a real CP cohort, we included ‘MRI compatible with the clinical picture’, as neuroimaging was one of the most significant factors for genetic CP. This is not unexpected given the higher yield of genetic findings in imaging negative CP cohorts.^4^, ^7^, ^10^ However, we believe normal imaging alone is not adequate in this decision making; instead, we believe the MRI findings must be compared with the clinical presentation^25^, ^26^, ^27^ and when the neuroimaging findings do not reflect the severity of motor impairment in combination with the comorbidities that the child has, then genetic testing should be considered. Therefore, we believe ‘MRI incompatible with clinical picture’ is a more useful term rather than ‘normal neuroimaging’ in decision making. We do acknowledge, however, that using a dichotomous approach as either compatible or not compatible, while pragmatic, may miss subtle findings.

Previous studies including a systematic review have shown a higher yield of genetic findings in children with CP plus intellectual disability, compared with those having CP alone,^1^, ^4^, ^7^, ^11^ which was also found in our study. Therefore, intellectual disability plus CP should prompt clinicians to consider genetic testing, as there is funding available for this indication in Australia. However, one of the limitations is the age at which intellectual disability can be confidently confirmed, which is typically older than 5 years when children are reclassified from having ‘developmental delay’ to intellectual disability.

Of the remaining differentiating factors, central hypotonia, microcephaly, and dysmorphic features were all significant in our cohort and are easily identifiable in children with CP and could be potentially applied at a young age (at CP diagnosis).

Central hypotonia often has a wide differential diagnosis and is common in genetic conditions. It is therefore not surprising that this factor was significantly associated with genetic CP. While we acknowledge that this remains controversial and not all communities accept hypotonic CP as a subgroup,^28^ if the child remains functionally impaired from a motor perspective, the diagnosis of hypotonic CP is acceptable in many countries including Australia, and does not negate the CP diagnosis.^29^, ^30^, ^31^ Regression of skills was significant in our retrospective GENE‐CP cohort. While regression would typically argue against a diagnosis of CP, which is by definition a static motor condition, we included any type of skill loss (motor, speech) reported by families and clinicians in this retrospective review. Although this is not something that is routinely reported in CP cohorts, family observations of ‘loss of skills’ or change in function were more common than expected. This would, however, be in keeping with CP being non‐progressive but not unchanging. An example from our cohort is a child we included as having ‘regression of skill’ who had unilateral CP and experienced an increase in falls following a growth spurt. Furthermore, neurodevelopmental disorders such as autism often co‐exist in children with CP, and can cause developmental regression; therefore these patients with regression were not excluded in our cohort if they also met criteria for CP.^32^, ^33^ We do acknowledge, however, that regression can also be found as part of a progressive disorder and, while this was not found in our cohort, this could also be a ‘red flag’ for a CP‐mimicker that requires a directed management pathway.^34^

Family history of similar phenotype, consanguinity, and early‐onset epilepsy were not significant in our genetic CP subgroup, which is probably because our cohort size was relatively small. Consanguinity is not common in Australia and there were no consanguineous families in our cohort. While seizures are common in children with CP (up to 30%), they can be due to both genetic and extrinsic (injury, infection) factors and were not differentiating in our study.

It is increasingly accepted that an early diagnosis of CP improves outcomes^35^ and we would add that early identification of genetic conditions may provide pathways for management during brain plasticity (repurposed or new). In this context, a staggered two‐tiered decision tree based on age may be required, whereby infants with an MRI brain scan not compatible with clinical presentation, dysmorphic features, hypotonia, microcephaly, and CP are considered promptly for genetic testing given these factors are identifiable early in infancy. A second tier of testing may be considered in those who are later diagnosed with developmental regression, intellectual disability, or show other unusual features such as deterioration with fasting/illness.

The limitations of this study were as follows. First, the 78 clinical and radiological variables that were tested in our cohort were generated by neurologists, rehabilitation physicians, and CP researchers who were expert in CP, on the basis of their understanding of the literature, and by comparing this with the international CP registries; there may have been important clinical and radiological factors missing from this process. Second, we performed an expert survey to determine what factors were thought to be indicative of a genetic cause of CP; however, a better method would have been to perform a Delphi study with multiple survey rounds to form a ‘consensus’ opinion. In addition to this, our expert panel, while international, lacked experts from low‐ and middle‐income countries, and future efforts should enable contributions from these countries to improve equitable access to clinical genomics globally. Third, our retrospective cohort study was performed in a tertiary hospital which may have enriched our cohort with atypical patients or complex patients, requiring more clinical care. Compared with community‐based cohorts, our tertiary cohort probably contained more children born at term, with hypotonia, mixed spasticity–dystonia, and children with atypical features such as regression. Regression is a particularly challenging phenomenon as, strictly speaking, patients with CP should not have loss of skills and regress. However, in real‐world medicine, patients with a CP phenotype can have loss of skills over time, or change in function, and hence our inclusion of this phenotype. Dyskinetic CP is less common (~15% CP) than spastic/hypertonic CP in most CP cohorts.^36^, ^37^ In our cohort, the proportion of children with pure spasticity was 30%, which is similar to a recently published study^38^ reporting spasticity in 36% but low compared with other CP cohorts which reported spasticity in up to 80%.^36^, ^37^ We think this was because, in our opinion, most children with spasticity also have evidence of dystonia. This mixed ‘spasticity–dystonia’ group may have been over‐represented in our cohort because we recruited from a tertiary hospital which has a movement disorder clinic and a deep‐brain stimulation service which could have enriched our cohort with more complex patients. It is clear from the literature there are factors that we know are more common in genetic causes of CP (dysmorphism, normal MRI, consanguineous biological parents, similar phenotype in other family members) and, while we reviewed expert opinion, unless we do a prospective assessment of factors and outcome, we will inherently be biased towards things that are known and established (confirmation bias).

A further limitation of the study was the absence of a neuroradiologist to re‐report on the imaging, although we used formal radiologists' reports. While neuroradiologists are easily accessible for research tasks in some tertiary centres, this is not true of our centre and most centres who look after children with CP around the world. Paediatric neurologists around the world have increasingly taken on the role of linking neuroradiological findings with clinical syndromes, and planning investigations including genomic testing for children with CP. Finally, as this was a retrospective cohort, not all children in the non‐genetic CP subgroup had genetic testing because genetic testing is not a standard pathway in CP and relies on the managing clinician's clinical suspicion. Furthermore, children who had a negative microarray/WES did not progress to WES/whole‐genome sequencing, which is becoming more widely available/accessible and may have increased the number of children with genetic CP. Review of the genetic variants and variant of unknown significance with a deeper genetic analysis and functional studies may have also increased the genetic findings as has been suggested in previous cohorts.^29^, ^39^, ^40^

Although most studies exploring CP genomics so far have been cohort studies, population‐based genomic studies have the ability to identify common vulnerability genes, involved in immune or metabolic function.^41^ A future prospective study from a community service that sees all patients with CP would reduce severity and confirmation bias. A prospective study has been approved by SCHN Ethics committee in our centre to assess the findings of this retrospective study. The current study, comparing imaging with the clinical picture, was facilitated by recruiting children up to 10 years. The accuracy of this factor when applied in children under 2 years is yet to be determined.

## CONCLUSION

Genetic conditions are broadening our understanding of why children have CP. Experts in CP and clinicians managing CP agree that genetic testing should be considered in the diagnostic work‐up for children with CP; however, establishing a cost‐effective clinical approach is needed. Our study has shown that there are factors in a child's clinical presentation that may improve the yield of genetic testing; however, further prospective studies on an undifferentiated CP cohort are needed to refine these.

## FUNDING INFORMATION

The Gene‐CP project was funded by a Cerebral Palsy Alliance Research Institute grant: Project Grant PRG00323. Funding was used for employment of a research assistant to help with the telephone interviews.

## Supporting information


**Appendix S1:** GENE‐CP Survey.

**Appendix S2:** GENE‐CP Expert Survey.

**Appendix S3:** Case examples: “MRI compatible and not compatible with clinical syndrome”.

**Table S1:** Factors tested in Expert survey and retrospective Cohort.

**Table S2:** Genetic variants identified in retrospective cohort.

**Figure S1:** Recruitment and breakdown of retrospective cohort based on genetic testing.

**Figure S2:** Expert survey and outcome of factors supportive and against a diagnosis of genetic CP.

## Data Availability

The data that support the findings of this study are available on request from the corresponding author. The data are not publicly available due to privacy or ethical restrictions.
