# Supplementary material for: Genetic testing in cerebral palsy with clinical and neuroimaging variables
Source: Dev Med Child Neurol. 2025 Apr 5;67(11):1443–52. doi: 10.1111/dmcn.16323 (PMC12521637; doi:10.1111/dmcn.16323)
Supplement: Supplementary file 1 — Appendix S1: GENE‐CP Survey. [file DMCN-67-1443-s002.pdf]

GENE-CP Survey

Start of Block: Intro & Demographics

Intro The purpose of this survey is to explore the current understanding and approach of genetic testing for people with CP. The current clinical approach to genetic testing of children with CP is unclear. There are no right or wrong answers, feel free to provide as much or as little information as you like. The survey will be anonymous and what you say will not be linked back to you personally. The information we obtain from the survey may be used to guide future focus-group discussions later.

Page Break

Q1 What is your current position?

- ○ Paediatrician (1)
- ○ Developmental Paediatrician (2)
- ○ Paediatric Neurologist (3)
- ○ Rehabiliation physician (5)
- ○ Others, please specify (6) ____

Q2 How long have you been working in your current position?

- ○ 1 to 5 years (1)
- ○ 6 to 10 years (2)
- ○ Over 10 years (3)

Q3 Is your clinical position..

- Hospital based (1)
- Private clinic (2)
- Mixture of hospital and private (3)
- Community based (4)

End of Block: Intro & Demographics

Start of Block: Perspectives

Q4 Clinically, do you see children with a diagnosis of Cerebral Palsy (CP)?

- Yes (1)
- No (2)

Q5 Approximately how many children/year do you see who have CP?

- less than 10/ year (1)
- 10 to 20/ year (2)
- more than 20/year (3)

Q6 As part of your role, do you investigate for causes of CP?

- Yes (1)
- No (2)

Page Break

Q7 Do you think genetic testing has a role in CP?

- Strongly agree (1)
- Somewhat agree (2)
- Neither agree nor disagree (3)
- Somewhat disagree (4)
- Strongly disagree (5)

Q8 In your personal opinion, do you think that if a child has a genetic diagnosis related to their neurological symptoms, then they cannot still have a clinical diagnosis of CP?

- Strongly agree (1)
- Somewhat agree (2)
- Neither agree nor disagree (3)
- Somewhat disagree (4)
- Strongly disagree (5)

Q9 What percentage of children with CP do you think may have a genetic cause of CP?

	0 10 20 30 40 50 60 70 80 90 100
Percentage of children with a genetic cause of CP ()	

Q10 In your own clinical practice, do you request genetic testing in children with CP?

- Yes (1)
- No (2)

Display This Question:

If In your own clinical practice, do you request genetic testing in children with CP? = Yes

Q11 If yes, what proportion of the children with CP that you see in your clinical practice undergo genetic testing of any type? (including chromosomal microarray, gene panel, exome, genome)

- None (1)
- less than 5% (2)
- 5 to 25% (3)
- 25 to 50% (4)
- more than 50% (5)

Q12 What (if any) would be the clinical features in a child with CP that would prompt you to consider genetic testing? List up to 5 findings.

____

Q13 What are (if any) the radiological features of a child's imaging that would influence your decision to proceed with genetic testing? List up to 5 findings.

____

____

____

____

Q14 If you have ordered genetic testing for some children with CP, which of the following tests have you ordered? (tick as many as applies)

- [ ] Chromosomal microarray (1)
- [ ] Individual gene sequencing (2)
- [ ] Gene panel (3)
- [ ] Exome sequencing (4)
- [ ] Gene panel off an exome background (5)
- [ ] Whole genome sequencing (6)
- [ ] Research testing (7)

Q15 If you have performed genetic testing for children with CP, what proportion did you find:

	0 10 20 30 40 50 60 70 80 90 100
positive test result / clear cut genetic diagnosis ()	
variant of uncertain clinical signficance ()	
negative test result/ no diagnosis ()	
incidental finding ()	
Click to write Choice 5 ()	

Page Break

Q16 Do you work with a genetic counsellor when offering genetic testing in children with CP?

- Yes (1)
- No (2)
- Not Applicable (3)

Q17 In your personal opinion, do you think that there is adequate information available to help you decide which children with CP maybe suitable for genic testing?

- Strongly agree (1)
- Somewhat agree (2)
- Neither agree nor disagree (3)
- Somewhat disagree (4)
- Strongly disagree (5)

Q18 Do you think genetic testing might be beneficial for the family of a child with CP?

- Strongly agree (1)
- Somewhat agree (2)
- Neither agree nor disagree (3)
- Somewhat disagree (4)
- Strongly disagree (5)

Q19 If so, in what ways (tick all that apply)

- ☐ reproductive counselling (1)
- ☐ connection with support group (2)
- ☐ relieving diagnostic odyssey (3)
- ☐ planning for delivery of future children (4)
- ☐ targeted treatment options (6)
- ☐ others, pls specify (5) ____

Q20 Do you think genetic testing can alter the clinical management of children with CP?

- ○ Strongly agree (1)
- ○ Somewhat agree (2)
- ○ Neither agree nor disagree (3)
- ○ Somewhat disagree (4)
- ○ Strongly disagree (5)

Q21 If so, in what ways? (tick all that apply)

- ☐ guidance on choice of medication (1)
- ☐ tailored therapy or drugs (2)
- ☐ interventions (3)
- ☐ other, please specify (4) ____

Q21 If you have children with CP that have a genetic diagnosis, how did the diagnosis impact your clinical management?

____

____

____

____

____

Q23 In your experience, how easy was it to access genetic testing for children with CP?

- ○ Extremely easy (1)
- ○ Somewhat easy (2)
- ○ Neither easy nor difficult (3)
- ○ Somewhat difficult (4)
- ○ Extremely difficult (5)

Q24 What, if any, are the main barriers to you accessing genetic testing?

____

____

____

____

____

Q25 In your opinion, what impact does this lack of access have on families?

____

____

____

____

____

Q26 Who do you think should be responsible for requesting genetic testing on children with CP?

____

____

____

____

____

End of Block: Perspectives
