# Supplementary material for: Genetic testing in cerebral palsy with clinical and neuroimaging variables
Source: Dev Med Child Neurol. 2025 Apr 5;67(11):1443–52. doi: 10.1111/dmcn.16323 (PMC12521637; doi:10.1111/dmcn.16323)
Supplement: Supplementary file 2 — Appendix S2: GENE‐CP Expert Survey. [file DMCN-67-1443-s004.pdf]

GENE-CP Expert Survey

Participant ID

Q1. What is your name?

Q2. What is your job title?

Q3. Which country are you located at?

- Australia
- Canada
- United States of America
- United Kingdom
- Taiwan
- Malaysia
- Spain
- New Zealand
- Israel
- Others, please specify

Q4. Are you a:

- [ ] Child Neurologist
- [ ] Adult and Child Neurologist
- [ ] Rehabilitation/ Neurodisability medical specialist
- [ ] Rehabilitation/Neurodisability allied health expert
- [ ] Clinical geneticist
- [ ] Paediatrician with neurology interest?

Q5. Do you think there are any historical, clinical or radiological features that are suggestive of monogenic causes of Cerebral Palsy (CP)? If so, would a GENE-CP scoring help in recognizing these?

Strongly Disagree	Partially Disagree	Neutral	Partially Agree	Strongly Agree
○	○	○	○	○

Q6. Does your research include:

- [ ] Genetics of Cerebral Palsy (CP) and CP mimics
- [ ] Phenotyping of CP and CP mimics
- [ ] Radiology of CP and CP mimics
- [ ] ________ Others, please specify

Q7. Do some patients with CP have a monogenic cause of their disorder?

- ○ Yes
- ○ No

Q8. If yes, in an 'unselected cohort of CP patients', what proportion of patients are **currently diagnosable** with a monogenic cause of CP?

Q9. What percentage of an 'unselected cohort of CP patients' will be diagnosable with a monogenic cause of CP *in the future?* (in %)

Associations with Monogenic Causes of CP

Q10. The following questions are in the context of a child with CP or suspected CP.

With regards to the patient's phenotype, how strongly do you feel the following phenotypic features are suggestive of a monogenic cause of CP, as opposed to an acquired brain injury cause of CP?

Q11. General phenotypic features

	Strongly Disagree	Partially disagree	Neutral	Partially Agree	Strongly Agree
Dysmorphic features	○	○	○	○	○
Congenital anomalies	○	○	○	○	○
Skull deformities including synostosis	○	○	○	○	○

Q12. Movement disorder phenotypic features

	Strongly Disagree	Partially disagree	Neutral	Partially Agree	Strongly Agree
Eye movement disorder	○	○	○	○	○
Paroxysmal movement disorder	○	○	○	○	○

	Strongly Disagree	Partially disagree	Neutral	Partially Agree	Strongly Agree
Fluctuating movement disorder (diurnal/nocturnal)	○	○	○	○	○
Stereotypies	○	○	○	○	○
Change in distribution of motor features (eg tone/hand dominance) over time	○	○	○	○	○

Q13. Movement disorder phenotypic features

	Strongly Disagree	Partially disagree	Neutral	Partially Agree	Strongly Agree
Dystonia in a term born infant	○	○	○	○	○
Dystonia in a premature born infant	○	○	○	○	○
A pure dystonic/dyskinetic phenotype	○	○	○	○	○
Diplegia in a term born infant	○	○	○	○	○
Diplegia in a premature infant	○	○	○	○	○

Q14. Movement disorder phenotypic features

	Strongly Disagree	Partially disagree	Neutral	Partially Agree	Strongly Agree
4-limbed spasticity in a term born infant	○	○	○	○	○
4-limbed spasticity in a premature infant	○	○	○	○	○
A mixed spastic/dystonic phenotype	○	○	○	○	○
A hemiplegic phenotype	○	○	○	○	○

	Strongly Disagree	Partially disagree	Neutral	Partially Agree	Strongly Agree
Dystonia with infantile epilepsy	○	○	○	○	○

Other neurological phenotypic features

Q15. The following questions are in the context of a child with CP or suspected CP.

With regards to the patient's phenotype, how strongly do you feel the following phenotypic features are suggestive of a monogenic cause of CP, as opposed to an acquired brain injury case of CP?

Q16. Other neurological phenotypic features

	Strongly Disagree	Partially disagree	Neutral	Partially Agree	Strongly Agree
Regression (loss of skills) of any neurological/developmental aspect	○	○	○	○	○
Areflexia	○	○	○	○	○
Sensory deficit	○	○	○	○	○
Optic atrophy	○	○	○	○	○
Hypertonia in the neonatal period	○	○	○	○	○

Q17. Other neurological phenotypic features

	Strongly Disagree	Partially disagree	Neutral	Partially Agree	Strongly Agree
Isolated or dominant ataxia	○	○	○	○	○

	Strongly Disagree	Partially disagree	Neutral	Partially Agree	Strongly Agree
Early onset epileptic encephalopathy (early infancy, eg Ohtahara or infantile myoclonic encephalopathy)	○	○	○	○	○
Autism	○	○	○	○	○
Normal or above average intellectual capacity	○	○	○	○	○
Intellectual disability	○	○	○	○	○

Q18. Other neurological phenotypic features

	Strongly Disagree	Partially disagree	Neutral	Partially Agree	Strongly Agree
Swallow/feeding out of proportion to motor limb deficits	○	○	○	○	○
The presence of epilepsy (regardless of CP type and MRI)	○	○	○	○	○
CP and epilepsy in the context of a normal MRI	○	○	○	○	○

Q19. Other neurological phenotypic features

	Strongly Disagree	Partially disagree	Neutral	Partially Agree	Strongly Agree
CP and autism in context of normal MRI	○	○	○	○	○
CP and the presence of learning disability in context of normal MRI	○	○	○	○	○
Presence of bilateral sensorineural hearing loss	○	○	○	○	○

	Strongly Disagree	Partially disagree	Neutral	Partially Agree	Strongly Agree
Presence of visual impairment	○	○	○	○	○

Patient History

Q20. With regards to the patient's history, how strongly do you feel each feature suggests a monogenic cause of CP, as opposed to as acquired brain injury cause of CP?

	Strongly Disagree	Partially disagree	Neutral	Partially Agree	Strongly Agree
A clinical history of birth asphyxia (low Apgar, poor cord pH plust need for resuscitation and admission to NICU)	○	○	○	○	○
Prematurity 23-28 weeks	○	○	○	○	○
Prematurity 29-36 weeks	○	○	○	○	○
Low birth weight (<3rd centile)	○	○	○	○	○
Small head circumference (<3rd centile)	○	○	○	○	○

Q21. With regards to the patient's history, how strongly do you feel each feature suggests a monogenic cause of CP, as opposed to an acquired brain injury cause of CP?

	Strongly Disagree	Partially disagree	Neutral	Partially Agree	Strongly Agree
A family history of similar motor phenotype	○	○	○	○	○
A family history of different motor phenotype	○	○	○	○	○
Family history of epilepsy	○	○	○	○	○

	Strongly Disagree	Partially disagree	Neutral	Partially Agree	Strongly Agree
Family history of ADHD/ASD/other neurodevelopment (non CP)	○	○	○	○	○
Parental consanguinity	○	○	○	○	○

Q22. With regards to the patient's history, how strongly do you feel each feature suggests a monogenic cause of CP, as opposed to an acquired brain injury cause of CP?

	Strongly Disagree	Partially disagree	Neutral	Partially Agree	Strongly Agree
Onset of movement disorder after 2 or more year period of normal development	○	○	○	○	○
Onset of non-movement disorder neurological features after a period of normal development	○	○	○	○	○
Neonatal seizures with HIE	○	○	○	○	○
Neonatal seizures outside of HIE context	○	○	○	○	○

Investigation Findings & Treatment Response

Q23. With regards to investigation findings, in the context of a child with CP, how strongly do you feel each feature suggests a monogenic cause of CP, as opposed to an acquired brain injury cause of CP?

	Strongly Disagree	Partially disagree	Neutral	Partially Agree	Strongly Agree
A normal MRI scan in the neonatal period	○	○	○	○	○

	Strongly Disagree	Partially disagree	Neutral	Partially Agree	Strongly Agree
A normal MRI scan between 1 month to <2 years of age	○	○	○	○	○
A normal MRI scan >2 years of age	○	○	○	○	○
A normal MRI scan at any age in a baby who was cooled for HIE	○	○	○	○	○
MRI showing arterial ischaemic stroke	○	○	○	○	○

Q24. With regards to investigation findings, in the context of a child with CP, how strongly do you feel each feature suggests a monogenic cause of CP, as opposed to an acquired brain injury cause of CP?

	Strongly Disagree	Partially disagree	Neutral	Partially Agree	Strongly Agree
MRI showing abnormality of the corpus callosum	○	○	○	○	○
An MRI with porencephaly	○	○	○	○	○
An abnormal MRI but not typical of HIE	○	○	○	○	○
An MRI scan with periventricular leukomalacia	○	○	○	○	○
An MRI typical of 'HIE-pattern of vascular injury'	○	○	○	○	○

Q25. With regards to investigation findings, in the context of a child with CP, how strongly do you feel each feature suggests a monogenic cause of CP, as opposed to an acquired brain injury cause of CP?

	Strongly Disagree	Partially disagree	Neutral	Partially Agree	Strongly Agree

	Strongly Disagree	Partially disagree	Neutral	Partially Agree	Strongly Agree
MRI showing intraparenchymal bleed	○	○	○	○	○
MRI with intraventricular bleed	○	○	○	○	○
An MRI with abnormal cerebellum or brainstem	○	○	○	○	○
MRI with predominant change in the bilateral globus pallidus	○	○	○	○	○
MRI with hypomyelination	○	○	○	○	○

Q26. With regards to investigation findings, in the context of a child with CP, how strongly do you feel each feature suggests a monogenic cause of CP, as opposed to an acquired brain injury cause of CP?

	Strongly Disagree	Partially disagree	Neutral	Partially Agree	Strongly Agree
MRI showing abnormal susceptibility in the basal ganglia/thalamus (iron deposition/calcification/bleed)	○	○	○	○	○
MRI with only T1 weighted hyperintensity in the basal ganglia/thalamus without T2 weighted signal change	○	○	○	○	○
MRI showing cerebellar atrophy/hypoplasia	○	○	○	○	○
MRI showing brainstem atrophy/hypoplasia	○	○	○	○	○
Sequential MRI showing atrophy of any brain region	○	○	○	○	○

Q27. With regards to investigation findings, in the context of a child with CP, how strongly do you feel each feature suggests a monogenic cause of CP, as opposed to an acquired brain injury cause of CP?

	Strongly Disagree	Partially disagree	Neutral	Partially Agree	Strongly Agree
MRI showing malformation of cortical development	◯	◯	◯	◯	◯
Abnormal CSF neurotransmitters	◯	◯	◯	◯	◯
Persistent lactic acidosis	◯	◯	◯	◯	◯
Abnormal CK	◯	◯	◯	◯	◯
Abnormal Lactate	◯	◯	◯	◯	◯

Q28. With regards to a patient's treatment response, how strongly do you feel each feature suggests a monogenic cause as more likely than acquired brain injury?

	Strongly Disagree	Partially disagree	Neutral	Partially Agree	Strongly Agree
Complete or near complete response to Levodopa	◯	◯	◯	◯	◯
Complete or near response to Deep Brain Stimulation	◯	◯	◯	◯	◯

Q29. Are there barriers to genetic testing for CP?

◯ No

◯ [ ] Yes, please specify

Q30. What access do your patients have to conduct exome sequencing for CP?

◯ government subsidized services

◯ private insurance

◯ personal patient funds

◯ research services

○ others, please specify

Q31. Are there any missing questions/factors you believe should be added?

Powered by Qualtrics
