# Supplementary material for: Genetic testing in cerebral palsy with clinical and neuroimaging variables
Source: Dev Med Child Neurol. 2025 Apr 5;67(11):1443–52. doi: 10.1111/dmcn.16323 (PMC12521637; doi:10.1111/dmcn.16323)
Supplement: Supplementary file 3 — Appendix S3: Case examples: “MRI compatible and not compatible with clinical syndrome”. [file DMCN-67-1443-s007.docx]

Case examples: “MRI compatible and not compatible with clinical syndrome”:

Case 1 : Full term born female infant. Her HC at birth was 33 cm (10^th^ centile) but she developed secondary microcephaly and by 12 months her HC was 42cm and below the 3^rd^ centile. Dystonic from birth and with evidence of contractures within first 6 months of life – both in upper and lower limbs. Difficulties with feeding led to severe failure to thrive and aspiration episodes. Required NGT and then PEG insertion by 24 months of age. The patient was diagnosed with cortical visual impairment from 12 months of age. MRI brain at 14 months show low lying cerebellar tonsils and tortuous optic nerves but no other abnormalities. The patient presented with febrile and afebrile seizures after the age of 14 months. Her clinical presentation was in keeping with spastic quadriplegic cerebral palsy. A trio exome was performed which showed a likely pathogenic mutation GRIN2a which was de novo. She has made very little developmental progress at age 5 years.

a.


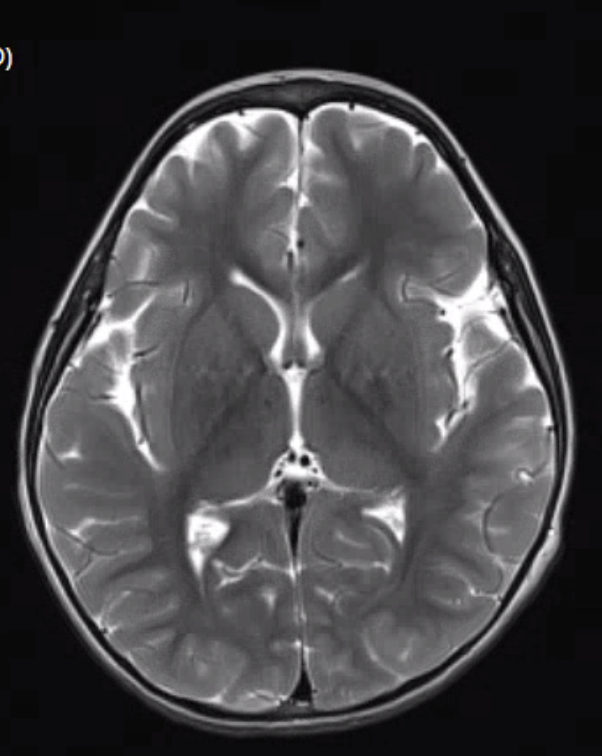

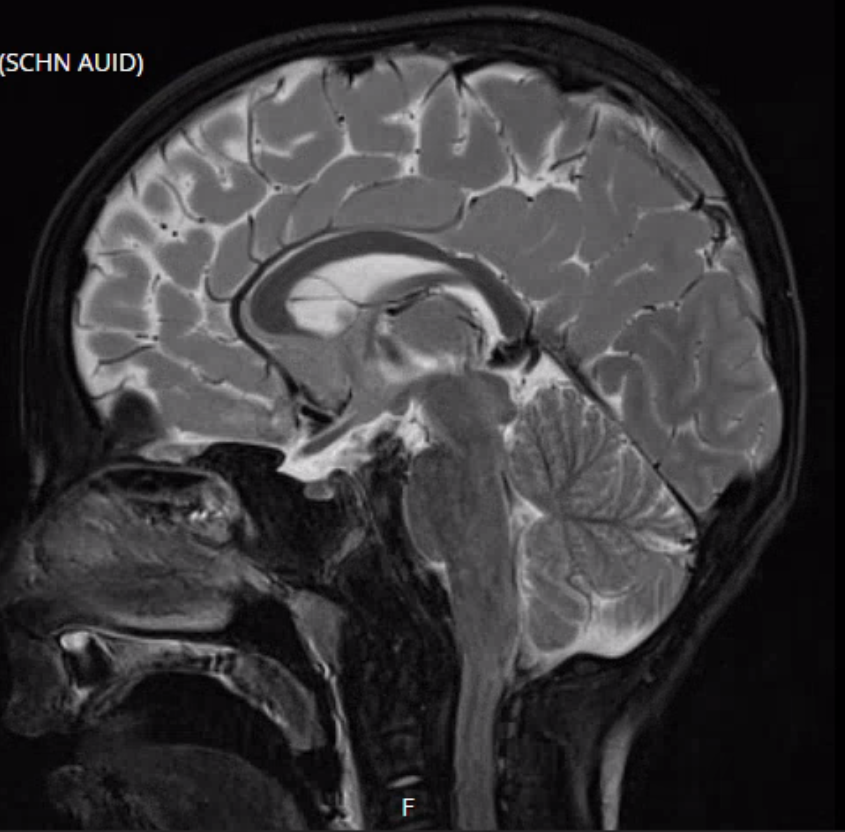


Figure 1 MRI brain age 5 years. Axial T2 on the left shows normal structures. Sagittal T2 on right shows low lying cerebellar tonsils.

***Application***: This child has 4 limb (spastic quadriplegic) CP with a normal MRI scan which shows her clinical picture is not in keeping with her presentation. This patient would be scored negative for ‘MRI compatible with the clinical picture’.

Case 2: Full term boy delivered by C-section for maternal fibroids. HC at birth and progress was on the 30^th^ centile, height and weight 2-5^th^ centile. At birth had a dusky episode presumed to be aspiration with his first bottle. Found to have a grade I IVH on the right, grade II on the left on HUS. MRI showed bilateral mild periventricular leukomalacia with a small cystic lesion in the occipital periventricular area on MRI performed at 14 months of age. The main concern was swallowing – he had frank aspiration and needed a nasogastric tube followed by PEG insertion at 18 months of age. He developed hand stereotypies at age 6 months with unusual postures of his upper limbs (when prone his wrists were internally rotated with fingers facing the chest and frequent flicking of fingers). He had poor eye contact at 6 months of age and would follow objects but not faces. He was diagnosed with severe speech delay. He presented with bilateral lower limb stiffness and diplegic posturing. He had a trio exome given his swallowing, social and speech issues were out of proportion to his MRI findings (clinical and radiological discordant). He was found to have a de novo mutation in AUTS2 gene.

***Application***: This child was referred to genetics because his clinical picture was not in keeping with his MRI changes. Whilst the mild diplegia which is consistent with PVL, however the severity of his swallowing and speech delay along with poor social interaction were not in keeping with the mild changes. The AUTS2 gene has been reported to also present with lower limb spasticity. This child was scored negative for “MRI compatible with the clinical picture”.

a. b.


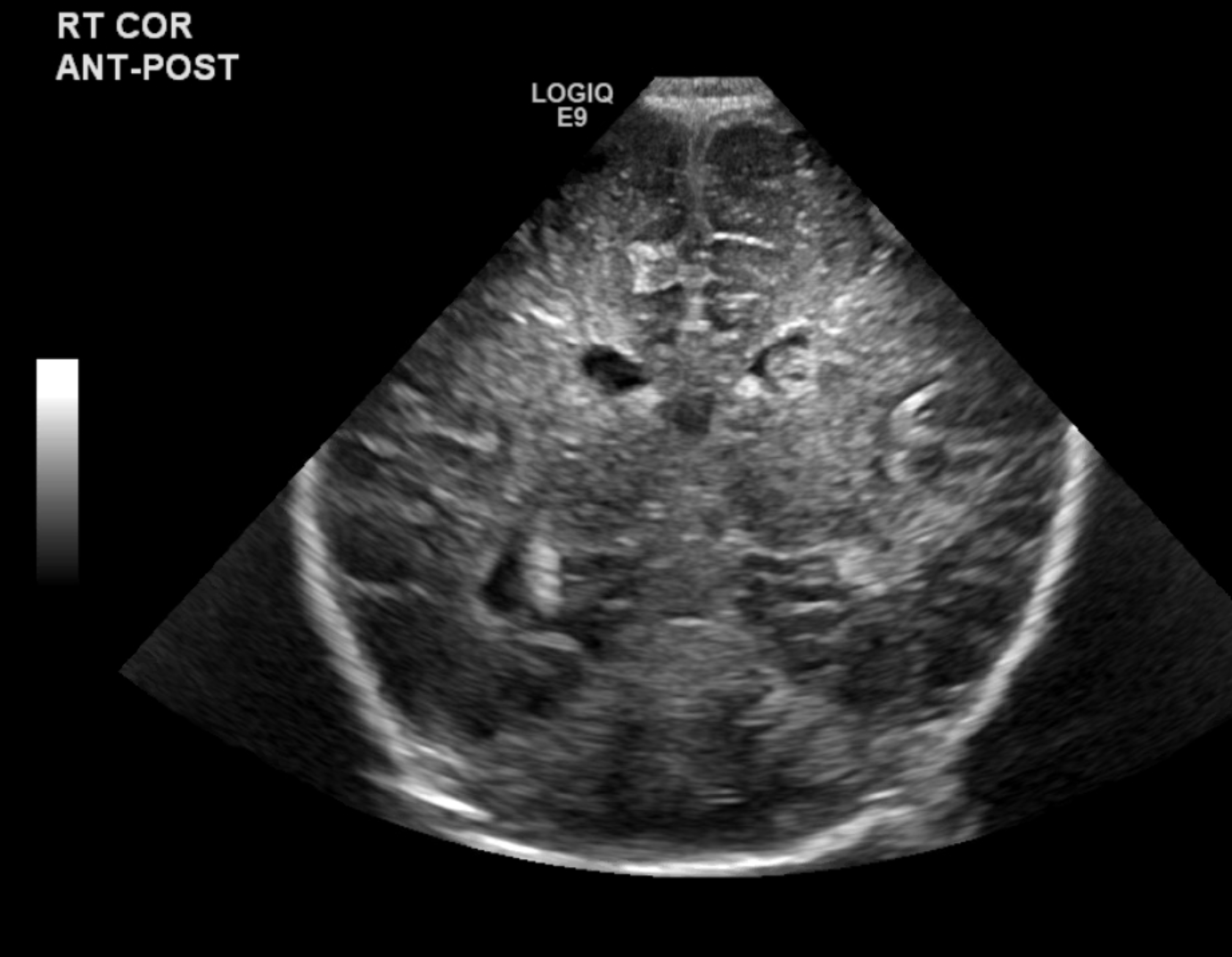

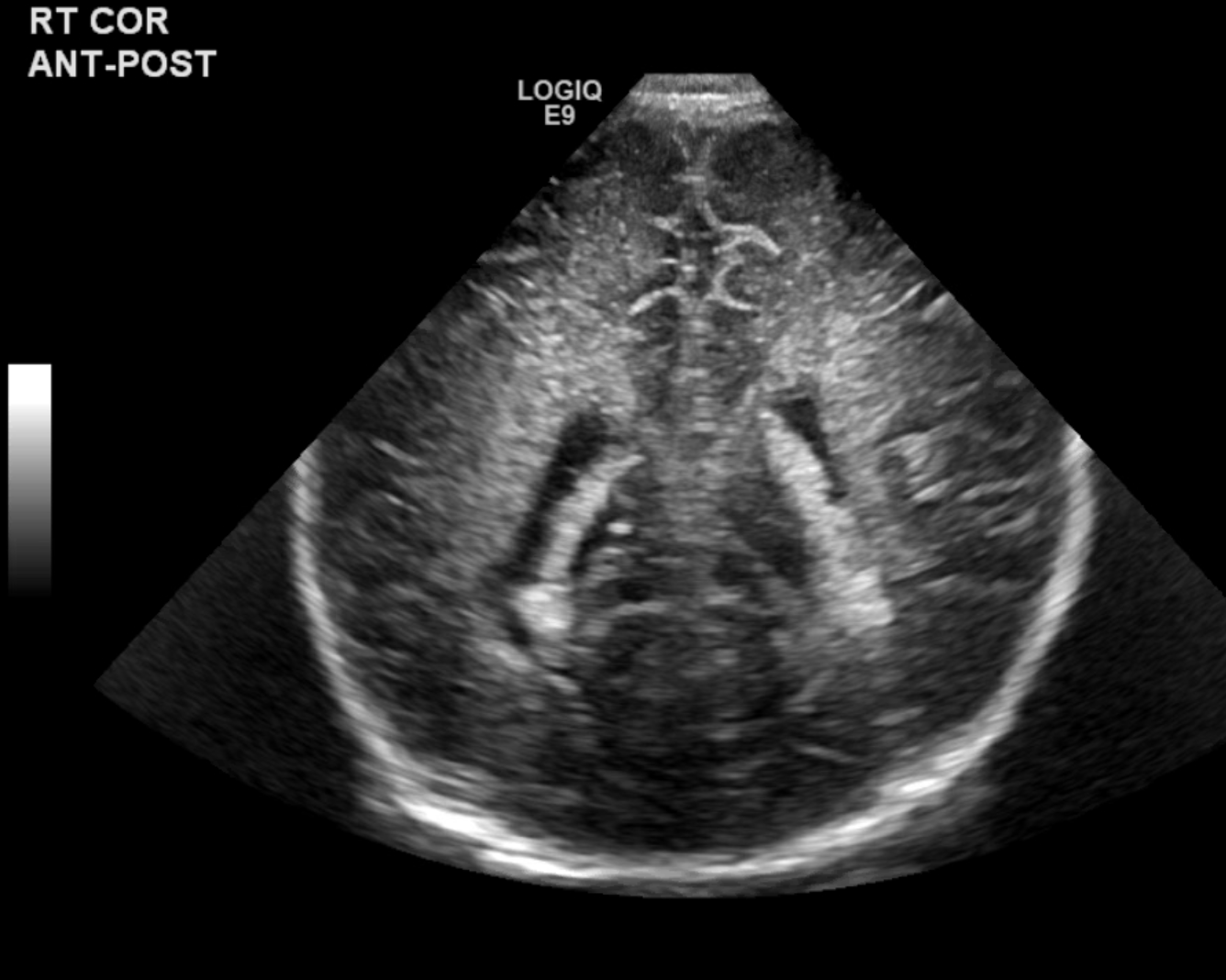


*Figure 2*(a and b) At birth HUS: Grade I IVH on right and II IVH on left.

1.
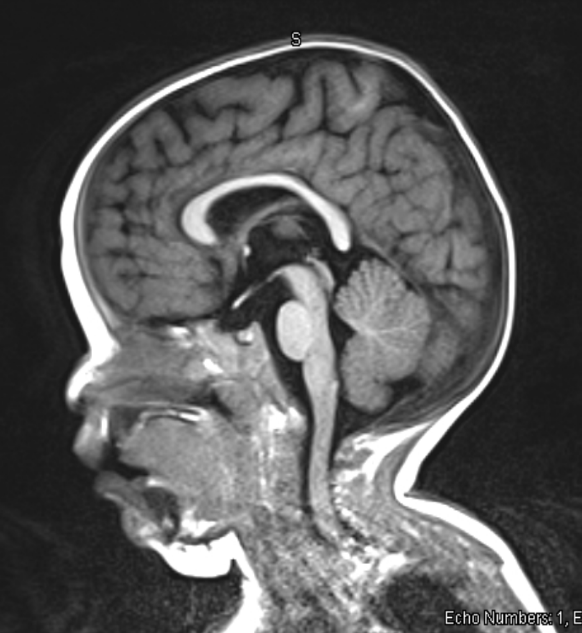
 b. c.


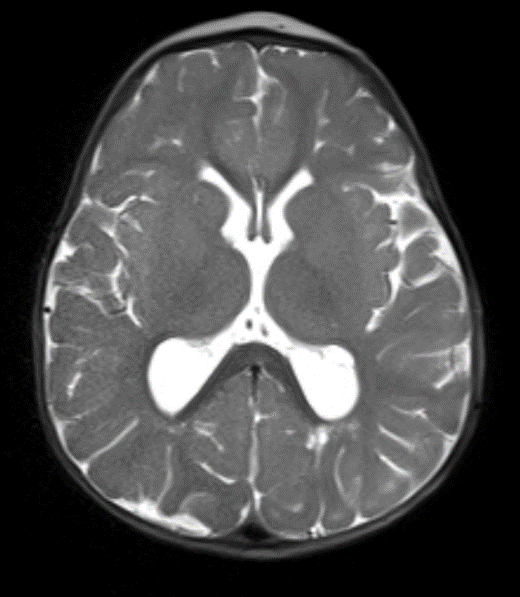

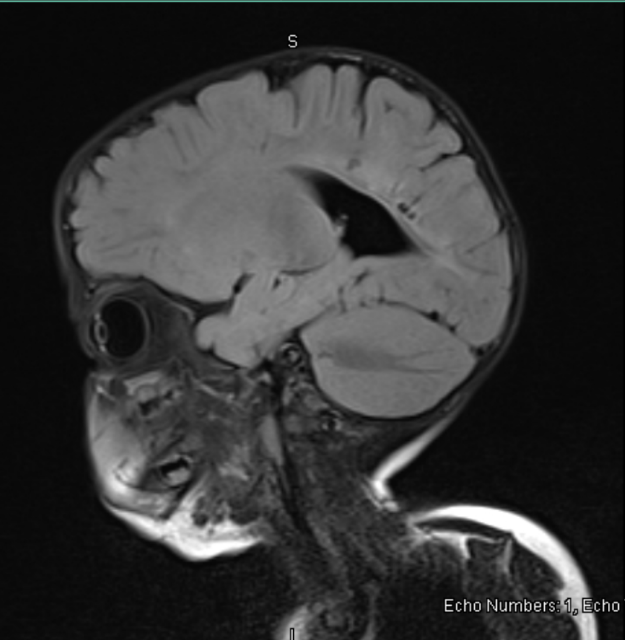


*Figure 3( a, b,c):* MRI brain at 14 months: sagittal FLAIR (a.) on the left shows posterior cystic PVL. T2 imaging on the right (b) shows mild ventricular dilation and small cystic lesion in occipital lobe and (c) Sagittal T1 FLAIR shows a mildly thinned corpus callosum.

Case 3: Full term male infant with BW 2.6kg and HC 31cm (both < 3^rd^ centile). Had true knot of umbilicus and the cord was wrapped around his neck. He was born with Apgar scores of 0,1,4 at 1,5,10 mins respectively. The patient needed intubation and resuscitation at birth. He met criteria for hypothermic cooling. He had multi-organ failure and neonatal seizures within the first 6 hours of birth which quickly resolved and were treated. He had an MRI within the first week of life which showed extensive bilateral hypoxic ischaemic changes with diffusion restriction of the basal ganglia, thalami, posterior limb of internal capsule and the peri-rolandic area. The patient recovered quickly from the multi-organ failure and was discharged in week 2 of life. He developed infantile spasms at 5 months of age with his EEG showing early evidence of epileptic encephalopathy in keeping with the severity of his brain injury. He remains on anti-seizure medications. His feeding has been intermittently an issue when he is unwell but generally he is taking all foods and fluids orally. He had a CGH microarray which was normal and a negative epileptic encephalopathy panel. At 13 months this child is presenting clinically as bilateral spastic quadriplegic cerebral palsy.

***Application***: This child has imaging that shows a severe injury in keeping with prolonged hypoxic changes and his clinical presentation is in keeping with these changes. He had a CGH microarray which was negative. He had the Invitae epileptic encephalopathy panel performed and it was negative. This child would be predicted to have a low yield genetic- CP diagnosis in the prospective study.

1. b.


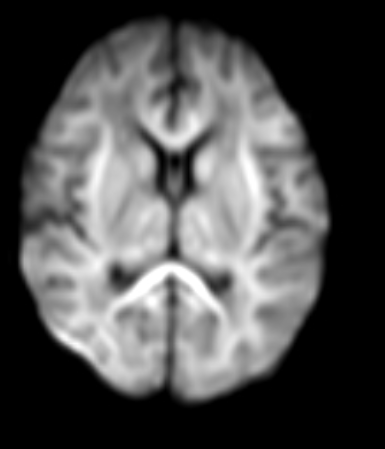

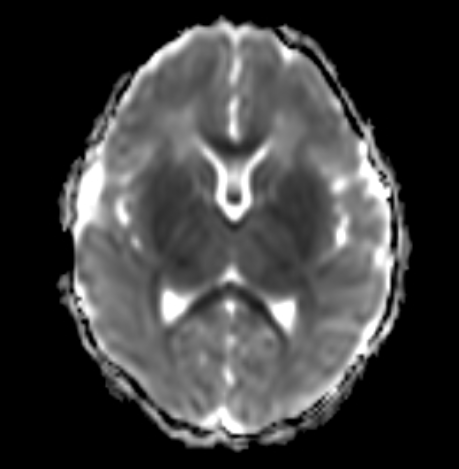


c. d.


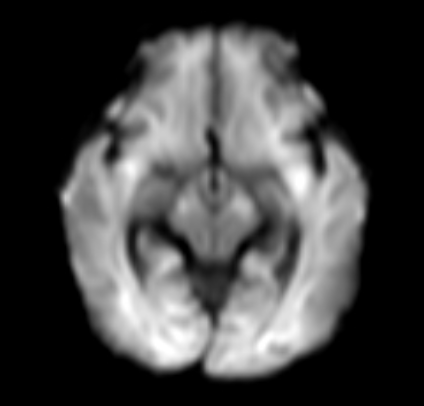

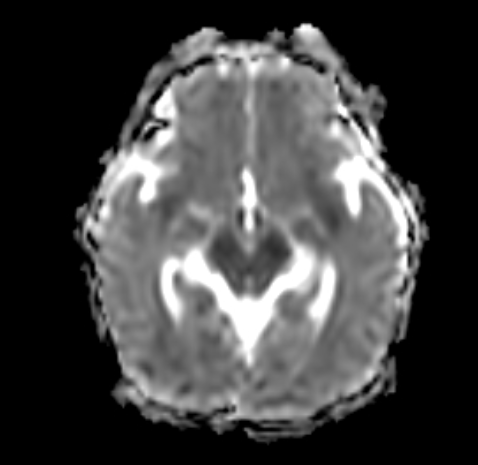


*Figure 4* (a,b,c,d): MRI brain performed day 5 of life following therapeutic cooling: DWI and ADC maps shows extensive diffusion restriction in the subcortical white matter, basal ganglia, thalamus (patchy) and cerebral peduncles.
