# Supplementary material for: Genetic testing in cerebral palsy with clinical and neuroimaging variables
Source: Dev Med Child Neurol. 2025 Apr 5;67(11):1443–52. doi: 10.1111/dmcn.16323 (PMC12521637; doi:10.1111/dmcn.16323)
Supplement: Supplementary file 4 — Table S1: Factors tested in Expert survey and retrospective Cohort. [file DMCN-67-1443-s001.pdf]

**Factors tested in Expert survey and retrospective Cohort**	
1	Complete or near complete response to Levodopa
2	Family history of a similar phenotype
3	Persistent lactic acidosis
4	Congenital anomalies
5	Dysmorphic features
6	Regression of any neurological or developmental aspect
7	Normal MRI >2 years of age
8	CP and epilepsy with a normal MRI
9	Early onset epileptic encephalopathy*
10	Onset movement disorder after >2 or more year period of normal development
11	Paroxysmal movement disorder
12	Parental consanguinity
13	Areflexia
14	Isolated dominant ataxia
15	Abnormal CSF neurotransmitters
16	MRI showing brainstem atrophy/hypoplasia
17	Fluctuant movement disorder (diurnal/nocturnal)
18	Onset of non movement disorder after a period of normal development
19	Abnormal lactate
20	CP and autism with a normal MRI
21	Family history of a different phenotype
22	MRI with abnormal cerebellum or brainstem
23	MRI showing cerebellar atrophy/hypoplasia
24	MRI showing malformation of cortical development
25	Pure dystonic dyskinetic phenotype
26	Abnormal CK
27	Normal MRI between one month to <2 years
28	CP and learning disability with a normal MRI
29	MRI with hypomyelination
30	Change in distribution of motor features over time*
31	MRI showing abnormal susceptibility in the basal ganglia and thalamus*
32	Complete or near response to Deep Brain Stimulation
33	Bouts of movement disorder exacerbations in sleep
34	Dystonia with infantile epilepsy
35	Skull deformities including synostosis
36	MRI T1 hypersensitivity in the basal ganglia and thalamus without T2 signal change
37	Sequential MRI showing atrophy of any brain region
38	Eye movement disorder
39	Optic atrophy
40	Normal MRI in the neonatal period
41	Hypertonia in the neonatal period

42	Diplegia in a term born infant
43	Family history of epilepsy
44	Neonatal seizures outside of HIE
45	MRI predominant change bilateral globus pallidus
46	Bilateral sensorineural hearing loss
47	MRI showing abnormality of the corpus callosum
48	Episodes of status dystonicus
49	Abnormal MRI but not typical of HIE
50	Dystonia in a premature infant
51	Swallowing/feeding out of proportion to motor limb deficits
52	Sensory deficit
53	Dystonia in a term born infant
54	Small head circumference (<3rd centile)
55	Family history of ADHD, ASD or other neurodevelopmental disorder
56	Presence of visual impairment
57	Mixed spastic dystonic phenotype
58	Normal MRI at any age for a baby cooled for HIE
59	Autism
60	Intellectual disability
61	Presence of epilepsy regardless CP type and MRI
62	4 limb spasticity in a term born infant
63	Stereotypies
64	4 limb spasticity in a premature infant
65	Low birth weight (<3rd centile)
66	Normal or above average intellectual capacity
67	MRI with porencephaly
68	Diplegia in a premature infant
69	MRI showing intraparenchymal bleed
70	A hemiplegic phenotype
71	Prematurity 29 to 36 weeks
72	MRI showing arterial ischaemic stroke
73	MRI showing intraventricular bleed
74	Neonatal seizures with HIE
75	MRI with periventricular leukomalacia
76	MRI typical of HIE pattern of vascular injury
77	Prematurity 23 to 28 weeks
78	Clinical history of birth asphyxia
