# Supplementary material for: Genetic testing in cerebral palsy with clinical and neuroimaging variables
Source: Dev Med Child Neurol. 2025 Apr 5;67(11):1443–52. doi: 10.1111/dmcn.16323 (PMC12521637; doi:10.1111/dmcn.16323)
Supplement: Supplementary file 5 — Table S2: Genetic variants identified in retrospective cohort. [file DMCN-67-1443-s005.pdf]

Supplementary Table 2 Genetic variants identified in retrospective cohort

ID	Analysis	Age (Y)	Sex	Primary Motor Phenotype	MRI N vs AbN	Gene(s)	Variant	Inheritance	Zygosity	ACMG Classification
8	MICROARRAY	3	M	Diplegic CP	AbN	NDE1, MYH11, ABCC1, ABCC6	c.16p13.12-p13.11	Unknown	Heterozygous	LP
10	MICROARRAY	4	F	Hypotonic CP	N	DLG4, ACADVL, CHRNB1, POLR2A	c.17p13.1	Unknown	Heterozygous	P
30	MICROARRAY	4	M	Mixed Spastic Dystonic Quadriplegic CP	AbN	AKT3	c.1393C>T; p.(Arg465Trp)	Unknown	Heterozygous	P
33	MICROARRAY	4	F	Left Dystonic Hemiplegic CP	N	TRRAP	7q22.1	Unknown	Heterozygous	P
53	MICROARRAY	8	F	Dystonic CP	AbN	ITGA6	c.2q31.1	Unknown	Heterozygous	P
56	MICROARRAY	10	M	Spastic CP	AbN	IFIH1 gene	C.2335C>T, p.Arg779Cys	De novo mutation	Heterozygous	P
63	MICROARRAY (antenatal)	5	F	Left Hemiplegic CP	AbN		c.1q21.1del	Paternal	Heterozygous	P
9	Trio WES	3	M	Spastic Dyskinetic Quadriplegic CP	N	MCT8 (Allan-Herndon Dudley Syndrome)	Performed overseas	Unknown	Hemizygous	P
11	Trio WES	6	M	Spastic diplegic CP	AbN	AUTS2	Single base deletion leading to a shift and introducing a premature stop codon.	De novo mutation	Heterozygous	P
12	Trio WES	6	M	Dystonic Quadriplegic CP	AbN	HDAC2 (Cornelia de Lange Syndrome)		Unknown		P
13	Trio WES	6	F	Dystonic Quadriplegic CP	AbN	GRIN2A	c. 1957A>G	De novo mutation	Heterozygous	P
14	Trio WES	5	F	Dyskinetic Quadriplegic CP	N	DDX3X	2 base pair deletion creates a frameshift resulting in a premature stop codon	De novo mutation	Heterozygous	P
18	Trio WES	7	M	Mixed Spastic Dystonic Diplegic CP	AbN	AP4M1	p.Tyr65Phefs*50	Paternal and Maternal	Homozygous	P
19	Trio WES	11	F	Mixed Spastic Dystonic Quadriplegic CP	N	UBA5	c.907T>C, c.1111G>A	Paternal	Compound Heterozygous and heterozygous	LP
27	Trio WES	8	M	Mixed Spastic Dystonic Quadriplegic CP	AbN	TUBA1A	c.521C>T	De novo mutation	Heterozygous	P
34	Trio WES	5	M	Ataxic Quadriplegic CP	AbN	NPC1	c.2819C>T, p.(Ser940Leu)	Unknown	Heterozygous	P
37	Trio WES	3	M	Left Hemiplegic CP with Hypotonia	AbN	CACNA1A	MIM601011	De novo mutation	Heterozygous	P
41	Trio WES	10	M	Spastic Diplegic CP	AbN	L1CAM	Point mutation	Maternal	Hemizygous	P
51	Trio WES	3	M	Ataxic CP with hypotonia	N	CACNA1A	Chr19(GRCh38): g.13261526C>T NM_001127222.2: c.4174G>A p.(Val1392Met)	De novo mutation	Heterozygous	P
32	MICROARRAY Trio WES	4	F	Hypotonic CP	AbNl	FOXG1	c.584G>C, p.(Arg195Pro)	Maternal	Heterozygous	P

55	MICROARRAY Trio WES	2	M	Dystonic CP with Hypotonia	N	WNK1<br><br>TCF20 gene	c.12p13.33<br><br>Chr22(GRC h38): g.42213048 del NM_005650. 4: c.2258del p.(Leu753Ar gfs*30)	De novo mutation	Heterozygous	P
