# Supplementary figures and images for: Genetic testing in cerebral palsy with clinical and neuroimaging variables
Source: Dev Med Child Neurol. 2025 Apr 5;67(11):1443–52. doi: 10.1111/dmcn.16323 (PMC12521637; doi:10.1111/dmcn.16323)

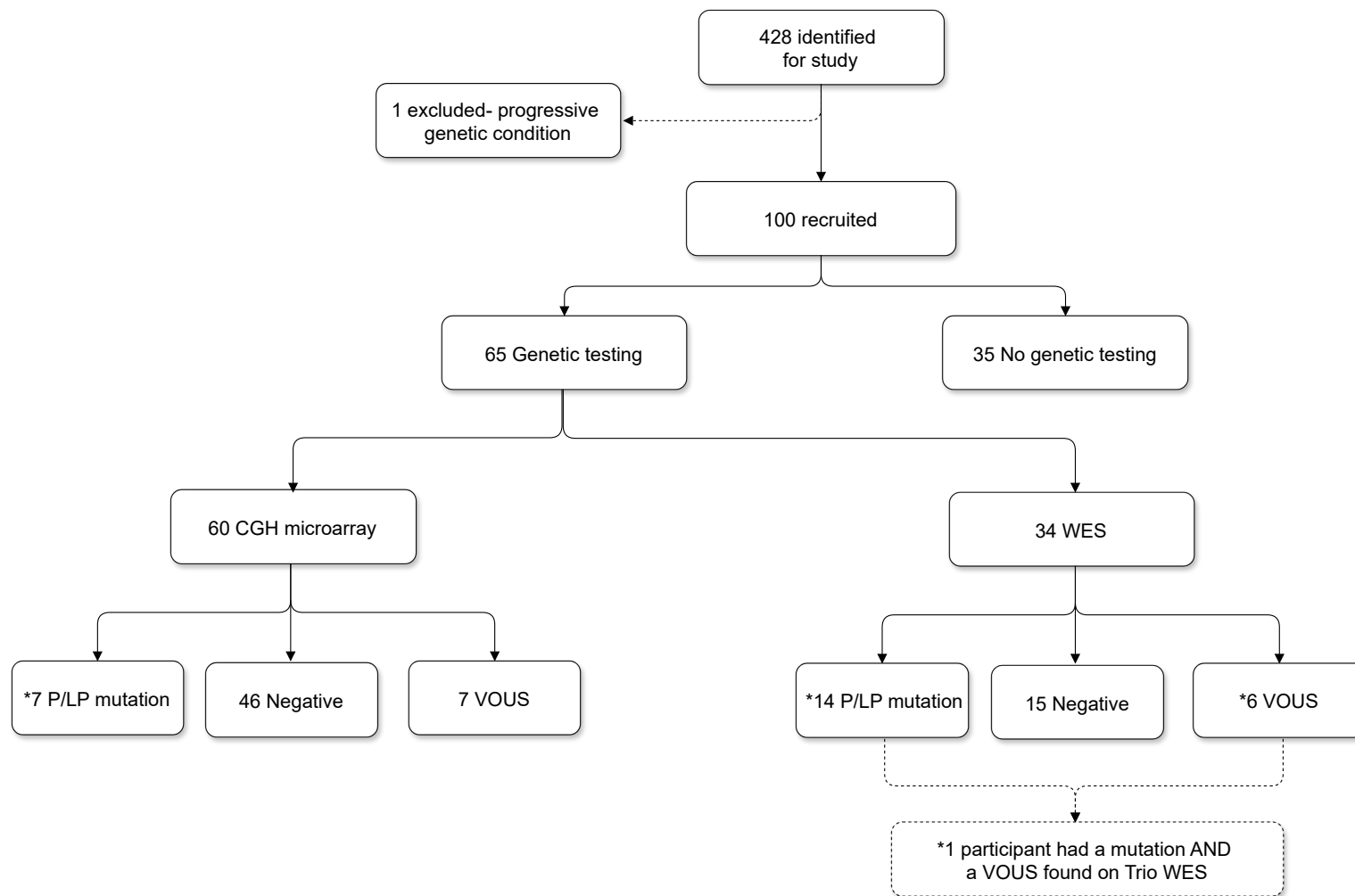

Supplement: Supplementary file 6 — Figure S1: Recruitment and breakdown of retrospective cohort based on genetic testing. [file DMCN-67-1443-s003.pdf]
