# Supplementary material for: Genetic testing in cerebral palsy with clinical and neuroimaging variables
Source: Dev Med Child Neurol. 2025 Apr 5;67(11):1443–52. doi: 10.1111/dmcn.16323 (PMC12521637; doi:10.1111/dmcn.16323)
Supplement: Supplementary file 7 — Figure S2: Expert survey and outcome of factors supportive and against a diagnosis of genetic CP. [file DMCN-67-1443-s006.pdf]

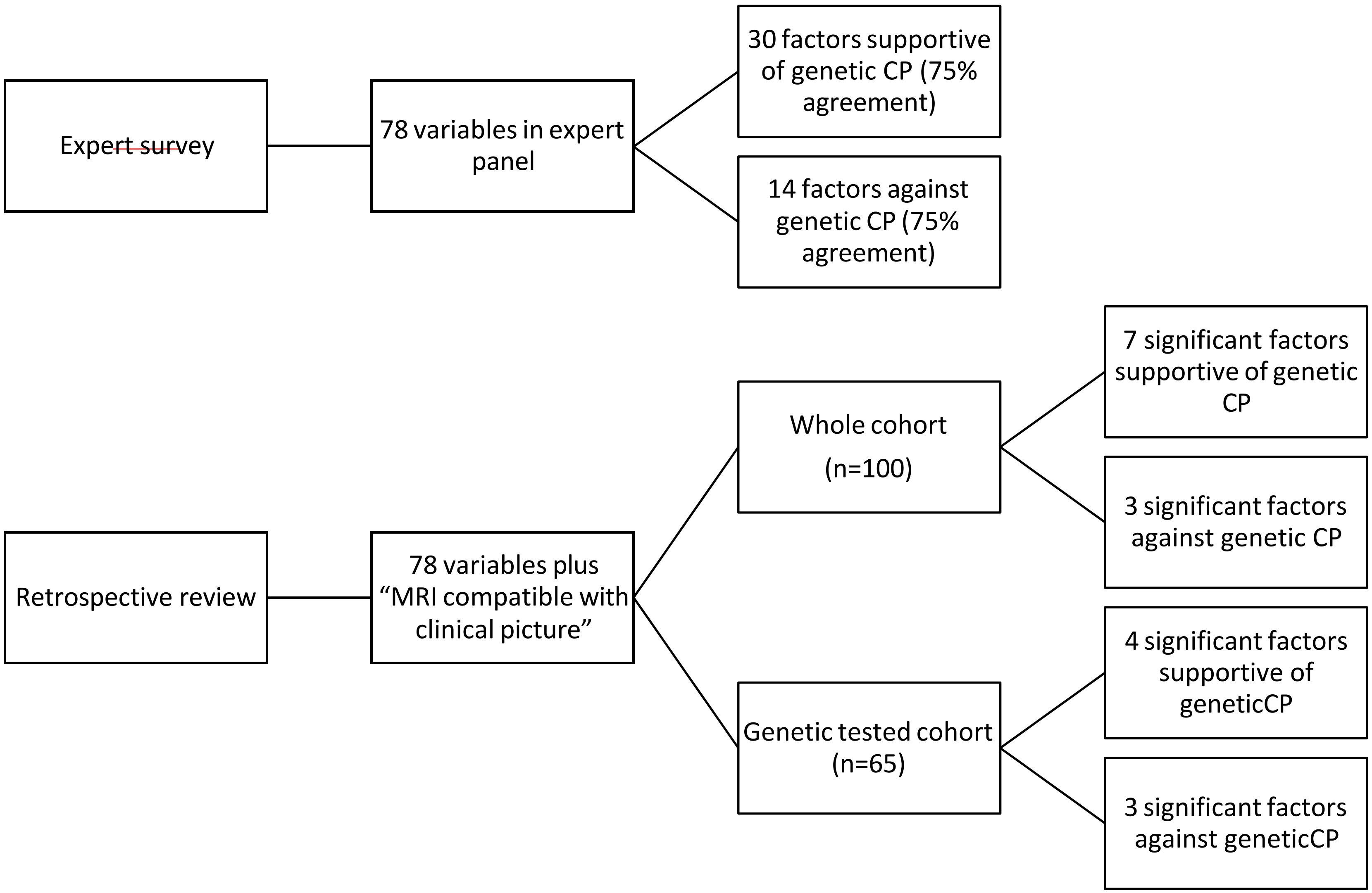

Expert survey and outcome of factors supportive and against a diagnosis of genetic CP. Retrospective review and testing of the variables on a cohort of 100 patients with CP with factors supportive and against genetic CP.
